# Pioglitazone Attenuates Vascular Fibrosis in Spontaneously Hypertensive Rats

**DOI:** 10.1155/2012/856426

**Published:** 2012-04-01

**Authors:** Dengfeng Gao, Ning Ning, Guanghua Hao, Xiaolin Niu

**Affiliations:** ^1^Department of Cardiology, The Second Affiliated Hospital, Xi'an Jiaotong University School of Medicine, Shaanxi, Xi'an 710004, China; ^2^Key Laboratory of Environment and Genes Related to Diseases of Ministry of Education, Xi'an Jiaotong University, Xi'an 710061, China; ^3^Department of Nuclear Medicine, The Second Affiliated Hospital, Xi'an Jiaotong University School of Medicine, Shaanxi, Xi'an 710004, China

## Abstract

*Objective*. We sought to investigate whether the peroxisome proliferator-activated receptor-*γ* (PPAR-*γ*) ligand pioglitazone can attenuate vascular fibrosis in spontaneously hypertensive rats (SHRs) and explore the possible molecular mechanisms. *Methods*. SHRs (8-week-old males) were randomly divided into 3 groups (*n* = 8 each) for treatment: pioglitazone (10 mg/kg/day), hydralazine (25 mg/kg/day), or saline. Normal male Wistar Kyoto (WKY) rats (*n* = 8) served as normal controls. Twelve weeks later, we evaluated the effect of pioglitazone on vascular fibrosis by Masson's trichrome and immunohistochemical staining of collagen III and real-time RT-PCR analysis of collagen I, III and fibronectin mRNA.Vascular expression of PPAR-*γ* and connective tissue growth factor (CTGF) and transforming growth factor-*β* (TGF-*β*) expression were evaluated by immunohistochemical staining, western blot analysis, and real-time RT-PCR. *Results*. Pioglitazone and hydralazine treatment significantly decreased systolic blood pressure in SHRs. Masson's trichrome staining for collagen III and real-time RT-PCR analysis of collagen I, III and fibronectin mRNA indicated that pioglitazone significantly inhibited extracellular matrix production in the aorta. Compared with Wistar Kyoto rats, SHRs showed significantly increased vascular CTGF expression. Pioglitazone treatment significantly increased PPAR-*γ* expression and inhibited CTGF expression but had no effect on TGF-*β* expression. *Conclusions*. The results indicate that pioglitazone attenuated vascular fibrosis in SHRs by inhibiting CTGF expression in a TGF-*β*-independent mechanism.

## 1. Introduction

Vascular fibrosis, characterized by excessive deposition of extracellular matrix (ECM) (e.g., collagen and fibronectin), is a major complication of hypertension and diabetes [1]. Connective tissue growth factor (CTGF) is a potent profibrotic factor implicated in pathologic fibrosis processes [2, 3]. In the cardiovascular system, CTGF is overexpressed in atherosclerotic lesions [4] and arteries of hypertensive animals [5]. In vascular smooth muscle cells (VSMCs), CTGF is involved in cell proliferation, migration, and apoptosis [6, 7]. Moreover, angiotensin II (Ang II) increases the production of CTGF and ECM, so CTGF is an intracellular mediator of hypertension-induced vascular fibrosis [8].

Thiazolidinediones (TZDs) such as pioglitazone are high-affinity ligands for peroxisome proliferator-activated receptor *γ* (PPAR-*γ*), a transcription factor of the nuclease hormone receptor superfamily [9]. TZDs are mainly used as insulin-sensitizing drugs in type 2 diabetes mellitus. They have potential roles in cardiovascular fibrosis [10]. *In vitro*, TZDs attenuate cardiovascular fibrosis by preventing ECM production and cell growth, thus inhibiting the inflammatory response and apoptosis in fibroblasts [11]. *In vivo*, TZDs could prevent cardiovascular fibrosis in rats with myocardial infarction [12] and Ang-II-infused and deoxycorticosterone, acetate-salt-treated rats [13, 14]. Whether TZDs could attenuate hypertension-induced vascular fibrosis and the possible mechanism have never been explored.

In this study, we observed the effects of pioglitazone on vascular fibrosis, and the expression of transforming growth factor *β* (TGF-*β*) and CTGF in spontaneously hypertensive rats (SHRs) to ascertain whether administering pioglitazone could attenuate vascular fibrosis with hypertension and to understand the underlying mechanisms.

## 2. Materials and Methods

 Our study was approved by the Institutional Animal Care Committee of Xian Jiaotong University and was conducted in accordance with the US National Institutes of Health Guide for the Care and Use of Laboratory Animals (Publication no. 85-23, revised 1996).

The experiments involved 8-week-old male SHRs and age-matched male Wistar Kyoto rats (provided by the Shanghai SLAC Laboratory Animal Technique Corp.).

### 2.1. Reagents

Pioglitazone was from Takeda Pharmaceuticals (Japan). Polyclonal anti-rat CTGF and TGF-*β* antibodies were from Santa Cruz Biotechnology (Santa Cruz, CA, USA). Rabbit polyclonal antibody against PPAR-*γ* was from Upstate Inc. (Chicago, IL, USA). DAB Horseradish Peroxidase Color Development Kit was from Beyotime (Suzhou, China), TRIzol and SuperScript III Platinum SYBR-Green Two-Step qRT-PCR kits were from Invitrogen (Carlsbad, CA, USA). DNA-free kit was from Ambion (Austin, TX, USA). Agarose gels were from Spanish Biochemicals (Pronadisa, Madrid). Reagents for enhanced chemiluminescence were from Pierce Corp. (Rockford, IL, USA).

### 2.2. Protocol

Normal WKY rats were the normal control group (*n* = 8). SHRs were randomly divided into 3 groups (*n* = 8 each) for treatment by oral gavage for 12 weeks: pioglitazone, 10 mg/kg/day [15]; hydralazine, 25 mg/kg/day; saline control. All rats were housed under similar conditions with a 12 hr light/dark cycle at 21 ± 1°C and humidity 55 ± 5%. Rats had free access to an ordinary diet and water.

Systolic blood pressure (SBP) was measured biweekly in conscious rats by tail-cuff plethysmography. Body weight was recorded every week throughout the study period.

At 12 weeks, rats were killed by intra-aortic administration of 10% potassium chloride and an excess amount of pentobarbital. One portion of the aorta was dissected and cleaned of fat and then frozen in liquid nitrogen for RNA extraction, and another portion was fixed in 4% formaldehyde solution, embedded in paraffin, and cut into sections, 4-5 *μ*m each, and, underwent trichromic Masson's trichrome and immunohistochemistry staining.

### 2.3. Real-Time RT-PCR

Total RNA was extracted by the use of TRIzol reagent, and DNA was removed by the use of the DNA-free kit. Real-time qRT-PCR with SYBR involved the use of the SuperScript III Platinum Two-Step qRT-PCR Kit on an ABI PRISM 7000 sequence detection PCR system (Applied Biosystems, Foster City, CA, USA). The primers used were previously reported [16]. Results were relative to the expression of glyceraldehyde-3-phosphate dehydrogenase (GAPDH) and were calculated by the 2^−∆∆CT^ method [17, 18].

### 2.4. Western Blot Analysis

Protein samples (20 *μ*g) were resolved on 10% SDS-PAGE, transferred to polyvinylidene difluoride membranes in a semidry system (Bio-Rad, Hercules, CA), and incubated with antibodies against CTGF (1 : 500), TGF-*β* (1 : 500), and *β*-actin (1 : 2000). Signals were revealed by chemiluminescence and visualized by exposure to X-ray films. Optical density was quantified with use of the Gel Doc 2000 system (Bio-Rad).

### 2.5. Immunohistochemistry

Paraffin-embedded rat thoracic aorta sections were incubated with primary antibodies against PPAR-*γ* (1 : 300) and collagen type III (Col III) (1 : 250) overnight at 4°C and then biotinylated and affinity-purified IgG (Zymed, USA) secondary antibody for 1 hr at 37°C. A streptavidin-enzyme conjugate was sequentially added for 20 min, and samples were incubated with substrate 3′,3′-diaminobenzidine (DAB), and then they underwent haematoxylin counterstaining. Negative control had no primary antibody. Quantitative analyses were using Qwin 550 quantitative image analysis system (Leica, German) by measuring the gray scale.

### 2.6. Statistical Analysis

Results are expressed as mean ± SD. Statistical significance between groups was assessed by one-way ANOVA, followed by post hoc Duncan multiple comparisons, with the use of SPSS v13.0 (SPSS Inc., Chicago, IL). A *P* < 0.05 was considered statistically significant.

## 3. Results

### 3.1. SBP in Rats

**Figure 1:**
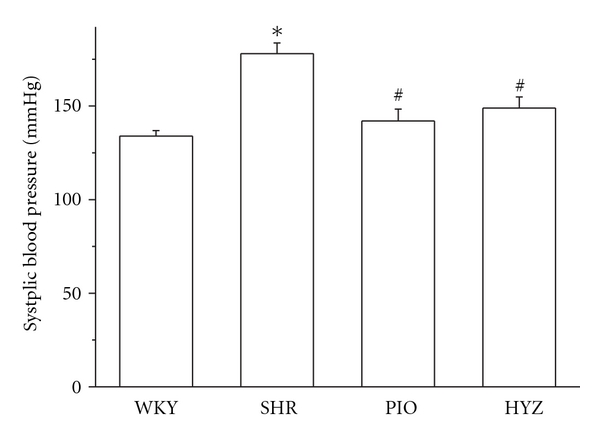
Systolic blood pressure in WKY and SHR rats after 12 weeks of treatment. WKY, Wistar Kyoto rats; SHR, spontaneously hypertensive rats; PIO, SHRs treated with pioglitazone; HYZ, SHRs treated with hydralazine. Values are mean ± SD. *P < 0.05 versus WKY; ^#^ *P* < 0.05 versus SHR.

At baseline and after treatment, SBP was higher in the SHR treatment group than in WKY rats (*P* < 0.05) (Figure 1). After treatment, SBP was significantly lower with pioglitazone and hydralazine treatment than in the SHR alone group (*P* < 0.05); the groups did not differ in body weight or heart rate at baseline or after treatment (data not shown).

### 3.2. Pioglitazone Attenuated ECM Expression in Thoracic Aorta

**Figure 2:**
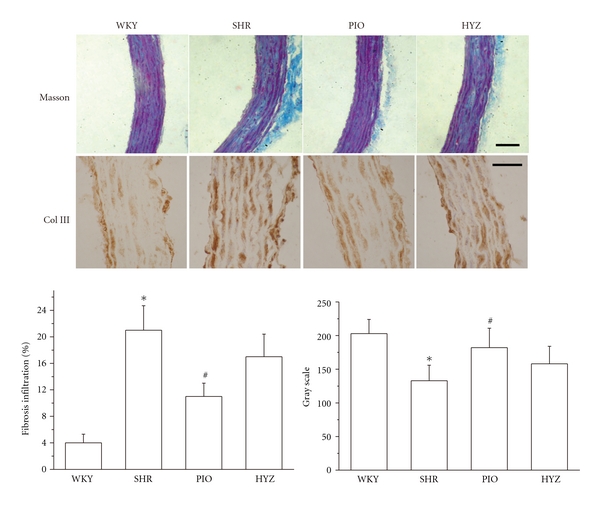
Masson's trichrome staining for fibrosis and immunohistochemical staining for collagen III and quantification (*n* = 6 rats, each group). Scale bar: 50 mm. Data are mean ± SD of 24 measurements in 6 slides. *P < 0.05 versus WKY; ^#^ *P* < 0.05 versus SHR.

**Figure 3:**
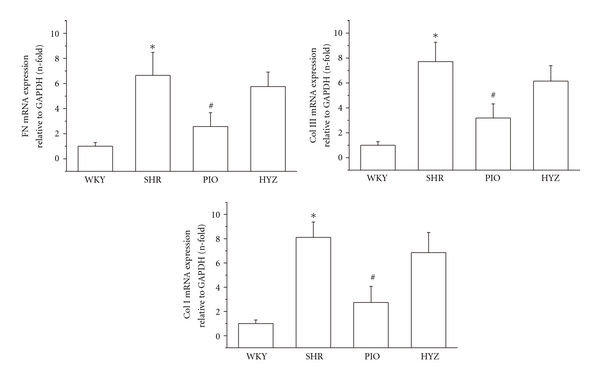
Effect of pioglitazone on collagen I, III and fibronectin mRNA expression. Data are relative to GAPDH expression and are mean ± SD of 3 experiments. *P < 0.05 versus WKY; ^#^ *P* < 0.05 versus SHR.

We then evaluated whether pioglitazone attenuated vascular fibrosis in SHRs. SHRs showed significant ECM deposition in thoracic aortas on Masson's trichrome and immunohistochemical staining of Col III (Figure 2). Real-time RT-PCR revealed increased fibronectin (FN), collagen I (Col I), and Col III mRNA expression in the aortic tissue of SHRs, which was significantly inhibited by pioglitazone treatment (Figure 3). These results indicated that pioglitazone but not hydralazine attenuated ECM deposition as compared with SHRs alone.

### 3.3. Vascular PPAR-*γ* Expression in Thoracic Aorta

**Figure 4:**
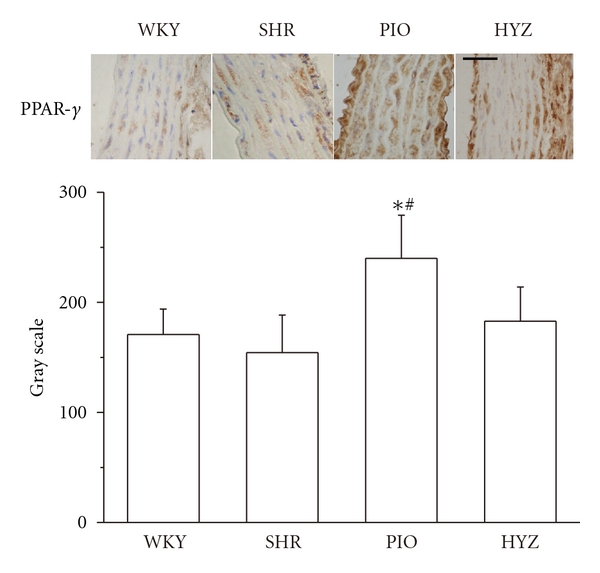
Immunohistochemical staining and quantification of peroxisome proliferator-activated receptor *γ* (PPAR-*γ*) in the thoracic aorta of rosiglitazone-treated and rosiglitazone-untreated rats after angiotensin II (Ang II) infusion (*n* = 6 rats each). Scale bar: 50 mm. *P < 0.05 versus WKY; ^#^ *P* < 0.05 versus SHR.

Pioglitazone treatment increased PPAR-*γ* protein expression as seen on immunohistochemistry (Figure 4) and was mainly located in nuclei of VSMCs, which indicates that PPAR-*γ* activation by pioglitazone may be involved in the suppression of ECM expression *in vivo*. Hydralazine had little effect on PPAR-*γ* expression.

### 3.4. Effect of Pioglitazone on CTGF and TGF-*β* Expression in Thoracic Aorta

**Figure 5:**
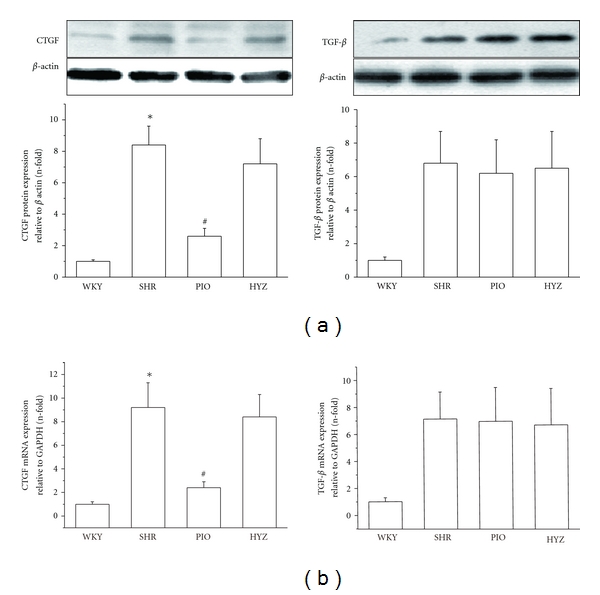
Effect of pioglitazone on mRNA and protein level of connective tissue growth factor (CTGF) and transforming growth factor *β* (TGF-*β*) in rat aortas (a) Western blot analysis of protein level of CTGF and TGF-*β*. *β*-actin was an endogenous control.  *P < 0.05 versus WKY; ^#^ *P* < 0.05 versus SHR (b) Real-time RT-PCR analysis of mRNA level of CTGF and TGF-*β*. Data are relative to GAPDH expression and are mean ± SD of 3 experiments. *P < 0.05 versus WKY; ^#^ *P* < 0.05 versus SHR.

 Real-time RT-PCR revealed increased CTGF mRNA expression in the aortic tissue of SHRs, which was significantly inhibited by pioglitazone treatment. Pioglitazone also attenuated CTGF protein expression (Figure 5(a)) but had no effect on TGF-*β* mRNA or protein expression in rat thoracic aortas (Figure 5(b)).

## 4. Discussion

Hypertension causes structural changes in the arteries (vascular remodeling) that involve alterations in cell growth, VSMC hypertrophy, and accumulation of ECM [19, 20]. Our data demonstrate that the PPAR-*γ* ligand pioglitazone can attenuate ECM production in the SHR aorta. Importantly, these effects are mediated in part by PPAR-*γ* activation likely through a TGF-*β*-independent pathway to inhibit CTGF expression. These findings provide novel evidence for the beneficial vascular effect of pioglitazone.

We previously showed that another PPAR-*γ* ligand, rosiglitazone, inhibited vascular fibrosis in Ang-II-infused rats [16]. In this study, we used SHRs, a typical rat model of hypertension, as a hypertension and vascular fibrosis model [10]. As expected, we found hypertension and vascular fibrosis in SHRs. Both pioglitazone and hydralazine treatment significantly lowered SBP, but only pioglitazone attenuated ECM production in the rat aorta. Pioglitazone has antihypertensive effects on other hypertensive models and hypertensive patients [21–24]. The novel finding that this agent reduced vascular fibrosis in SHRs may be explained by a direct action of pioglitazone on the vessel wall. BP lowering does not appear to play a role, because hydralazine decreased BP without any effect on vascular fibrosis and CTGF or TGF-*β* expression. These *in vivo *data confirm and extend results from our previous *in vitro *studies in which PPAR-*γ* ligands were shown to downregulate angiotensin-induced ECM production in VSMCs [16].

CTGF has been postulated to be involved in conditions involving overgrowth of connective tissue cells (e.g., systemic sclerosis, cancer, fibrotic conditions, and atherosclerosis). Our previous study confirmed that PPAR-*γ* ligand inhibited CTGF overexpression in response to Ang II in cultured VSMCs [16]. In the present study, treatment with pioglitazone for 12 weeks in SHRs significantly reduced vascular CTGF expression and markedly alleviated fibrosis infiltration. Thus, inhibiting vascular CTGF expression to inhibit vascular fibrosis may be a mechanism of the beneficial effects of pioglitazone on vascular fibrosis in SHRs.

In vascular fibrosis, TGF-*β* participates in regulating CTGF and ECM production [25]; importantly, the CTGF promoter has a TGF-*β* binding element [26], so we tested whether reduction of CTGF by pioglitazone was due to downregulated TGF-*β* expression. TGF-*β* expression in SHR aortas was not affected by pioglitazone, so pioglitazone attenuated CTGF expression by a TGF-*β*-independent mechanism. Some recent studies support our results. Pioglitazone attenuated left ventricular hypertrophy and CTGF expression without affecting TGF-*β* expression in stroke prone SHR [27]. Ang II may induce CTGF production [28] and vascular fibrosis [29] by a TGF-*β*-independent mechanism. Moreover, in human kidney fibroblasts, pioglitazone inhibited cell growth and reduced matrix production in a TGF-*β*-independent way [30]. Therefore, PPAR-*γ* may affect TGF-*β*-independent or post-TGF-*β* signals in regulating CTGF expression. Our previous studies indicated PPAR-*γ* activation downregulated CTGF expression via interaction with Smads and JNK pathways [16, 31]. Recently, Kim et al. [32] showed that another PPAR-*γ* ligand, rosiglitazone, inhibited CTGF and ECM production through the mTOR-P70S6 kinase and 4EBP1 pathways in vascular smooth muscle cells. So we think pioglitazone-modulated CTGF may mainly through pathways such as Smads, JNK, mTOR-P70S6 kinase, and 4EBP1.

In summary, the present study demonstrates that the PPAR-*γ* ligand pioglitazone attenuated vascular fibrosis in SHRs with hypertension by inhibiting CTGF expression by a TGF-*β*-independent way. These observations point to a potential mechanism of pioglitazone in preventing vascular fibrosis.
